# Bridging mechanistic evidence and clinical perspectives: traditional Chinese medicine in Menière's disease

**DOI:** 10.3389/fneur.2026.1915230

**Published:** 2026-10-20

**Authors:** Shuting Ye, Zhenhua Zhu

**Affiliations:** The First Affiliated Hospital of Hunan University of Chinese Medicine, Changsha, Hunan, China

**Keywords:** aquaporins, endolymphatic hydrops, Ménière's disease, traditional Chinese medicine, vestibular compensation

## Abstract

Ménière's disease (MD) is a debilitating inner-ear disorder characterized by recurrent vertigo, fluctuating sensorineural hearing loss, tinnitus, and aural fullness, with endolymphatic hydrops serving as its pathological hallmark. Conventional management, which includes comprising lifestyle modifications, pharmacotherapy, and surgical interventions for refractory cases and often yields limited efficacy and carries risk of adverse effects, underscoring the imperative for safer, more effective therapeutic strategies. Traditional Chinese Medicine (TCM), encompassing herbal phytotherapy and acupuncture, holds a rich clinical history in managing vertiginous conditions and is increasingly scrutinized within contemporary biomedical research. This review comprehensively reviews the mechanistic foundations and therapeutic utility of TCM in MD, aiming to establish it as a scientifically grounded component of personalized, evidence-based integrative management. We synthesize clinical and experimental evidence linking TCM interventions to core pathophysiological pathways in MD, specifically the regulation of molecular fluid transport, modulation of neuroinflammatory signaling cascades, enhancement of inner-ear microcirculation, and promotion of neuroplastic processes. Mechanistically, specific herbal formulations modulate aquaporin expression to restore endolymphatic fluid homeostasis, suppress MAPK and NF-κB-mediated inflammatory activity, improve cochleovestibular blood flow, and attenuate oxidative stress and apoptosis. Clinically, acupuncture has been associated with alleviation of symptom severity, accelerates central vestibular compensation, and preserves auditory and vestibular function. Integrating these insights, we propose a multi-tiered mechanistic model wherein TCM exerts immediate anti-inflammatory and fluid-regulatory effects, facilitates medium-term vestibular compensation, and provides long-term neuroprotection to potentially modify disease progression. By relating mechanistic insights to clinical data, this review highlights TCM as a promising adjunctive modality that warrants further rigorous investigation, offering a framework to inform mechanism-oriented treatment strategies and guide future translational and clinical research. Throughout the manuscript, we explicitly distinguish experimental evidence, mechanistic hypotheses, and human clinical evidence to facilitate critical appraisal.

## 1 Introduction

Inner ear disorders characterized by vertigo and equilibrium deficits impose a profound socioeconomic burden and severely compromise patient quality of life (1, 2). Among these pathologies, Ménière's disease (MD) Among these disorders, Ménière's disease represents a clinically important and uniquely debilitating inner-ear condition. Endolymphatic hydrops (EH), characterized by abnormal distension of the membranous labyrinth caused by excessive accumulation of endolymph, is considered a characteristic pathological feature associated with MD; however, it is neither specific to MD nor sufficient alone for diagnosis (3, 4). The clinical expression of MD is highly heterogeneous; vertigo attack frequency, duration, and audiometric configurations vary remarkably among individuals, underpinned by divergent etiological contributors (5, 6). This pronounced heterogeneity confounds diagnostic classification and hampers individualized therapeutic selection. Although contemporary consensus criteria have refined diagnostic precision by establishing strict thresholds for definite vs. possible cases, requiring two or more spontaneous vertigo episodes lasting between twenty min and twelve h coupled with documented fluctuating sensorineural hearing loss, the translation of these clinical phenotypes into mechanism-guided interventions remains challenging (5, 7). Consequently, rigorous patient stratification is imperative to mitigate heterogeneity in both clinical management and translational research.

Far from being a mere static bystander, endolymphatic hydrops represents the pivotal mechanistic link translating inner ear homeostatic disruption into the clinical phenotype of MD (8–10). Historically, the investigation of endolymphatic hydrops was constrained to postmortem temporal bone histopathology, a logistical limitation that severely bottlenecked translational advancements. However, recent breakthroughs in neuroimaging have revolutionized this landscape, enabling *in vivo* visualization and noninvasive, objective grading of hydrops. Delayed gadolinium enhanced magnetic resonance imaging of the inner ear now delivers reliable quantitative metrics for longitudinal monitoring, facilitating the systematic correlation of hydrops severity with the kinetics of hearing loss and vertigo recurrence (11, 12). These radiological advances bridge a long standing translational gap, illuminating a multifactorial pathogenic architecture (3, 6). It is now recognized that genetic susceptibility, viral insults such as herpes simplex virus, autoimmune cascades, and autonomic dysregulation converge to disrupt the delicate equilibrium between endolymph production and resorption. This pathogenic complexity underscores why conventional therapies targeting a single pathway so frequently yield inconsistent and suboptimal efficacy in real world clinical practice.

Current therapeutic paradigms for Meniere disease adopt a stratified approach tailored toward three primary objectives: aborting acute episodes, reducing attack frequency, and preserving residual cochleovestibular function. First line conservative regimens typically mandate dietary sodium restriction coupled with diuretics and betahistine (13–16). While designed to attenuate endolymphatic fluid retention and augment inner ear microcirculation, the clinical evidence supporting these measures remains equivocal, and patient responses are markedly fragmented. For patients refractory to conservative care, intratympanic interventions are deployed as second line modalities (17, 18). Intratympanic corticosteroids aim to suppress localized neuroinflammation, whereas intratympanic gentamicin induces chemical ablation of vestibular hair cells; however, the latter achieves vertigo control at the profound risk of permanent ototoxicity. For intractable, disabling cases, surgical interventions represent a definitive last resort. These range from hearing preservation strategies, such as endolymphatic sac decompression, to destructive operations, including vestibular nerve section and labyrinthectomy (19). Because the efficacy of sac decompression remains highly controversial, contemporary management struggles to strike an optimal balance between safety, efficacy, and patient compliance. This therapeutic impasse underscores the urgent need for multitargeted interventions capable of restoring systemic inner ear homeostasis.

Traditional Chinese medicine (TCM) rests on holistic principles and is implemented through herbal therapy and acupuncture. Preclinical studies suggest that TCM interventions may influence several pathological processes relevant to MD, including regulation of water transport proteins such as aquaporins, modulation of inflammatory signaling pathways including NF-κB, improvement of inner-ear microcirculation, and attenuation of oxidative stress-related injury (20, 21). Clinical integration of TCM in MD care is currently limited by a sparse evidence base (22, 23). Few studies apply explicit diagnostic criteria or patient subtyping; randomized controlled trials with adequate blinding and appropriate controls are scarce; objective endpoints such as delayed gadolinium-enhanced magnetic resonance imaging-based grading of hydrops and standardized audiometry are infrequently adopted; and long-term follow-up data remain limited. Moreover, the placebo response in MD can be substantial, with reductions in vertigo attack frequency of approximately fifty percent observed in placebo arms, a factor that must be carefully weighed when interpreting uncontrolled studies.

This review summarizes current clinical and preclinical evidence regarding TCM interventions in MD, examines their potential mechanisms in relation to established disease pathways, and discusses methodological priorities required for future translational research. Ultimately, this work aims to define the potential role of TCM within an evidence-based integrative framework while acknowledging the preliminary nature of existing evidence and the need for further high-quality clinical validation.

## 2 Pathophysiological mechanisms underlying MD

The pathogenesis of MD is multifactorial, with diverse proposed initiating factors including genetic susceptibility, viral infections, and dysregulated autoimmune responses (10, 14, 24). A pivotal concept emerging from converging evidence is that these disparate upstream triggers ultimately activate a limited repertoire of interconnected downstream pathways responsible for the development of EH and its associated clinical symptomatology (25). This paradigm of convergent pathogenic mechanisms elucidates the frequently observed limited efficacy of conventional therapies targeting a single pathway. Consequently, this pathophysiological framework provides a compelling rationale for therapeutic strategies capable of simultaneously modulating multiple shared pathological nodes, thereby potentially yielding synergistic therapeutic effects. Figure 1 provides a schematic overview of these convergent mechanisms and the key pathological nodes detailed in the following subsections.

**Figure 1:**
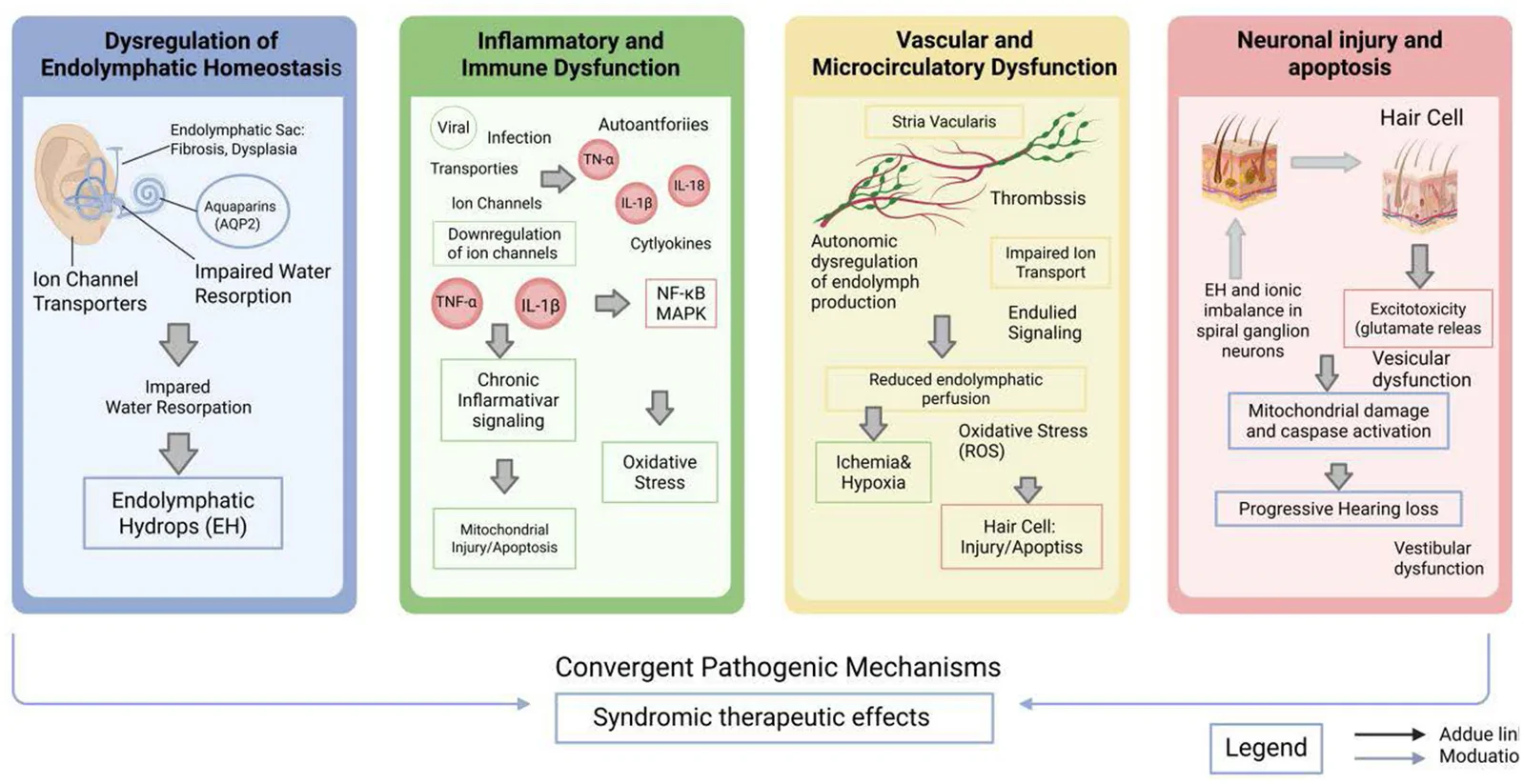
Schematic of convergent pathophysiological mechanisms in Menière's disease. Four interconnected domains are depicted: dysregulation of endolymphatic homeostasis, inflammatory and immune dysfunction, vascular and microcirculatory impairment, and neuronal injury with apoptosis. Impaired ion transport and aquaporin mediated water resorption in the endolymphatic sac favor endolymphatic hydrops. Viral exposures, autoantibodies and elevated proinflammatory cytokines such as tumor necrosis factor alpha and interleukin one beta activate NF kappa B (NF-κB) and MAPK signaling, sustaining chronic inflammation and oxidative stress. Microvascular dysregulation reduces cochleovestibular perfusion, precipitating ischemia, hypoxia and reactive oxygen species generation. Mechanical pressure and excitotoxic glutamate release, together with mitochondrial compromise, converge on caspase dependent apoptosis of hair cells and spiral ganglion neurons. Bidirectional arrows denote cross talk among domains and illustrate shared downstream nodes. The figure emphasizes why single target therapies often fall short and provides a rationale for multifaceted interventions that simultaneously modulate ion and fluid balance, immune responses, microcirculation and neuronal survival.

### 2.1 Dysregulation of endolymphatic homeostasis: ion channels, aquaporins, and the endolymphatic sac

The maintenance of normal auditory and vestibular function is critically dependent on a tightly regulated balance of ionic composition and fluid volume between endolymph and perilymph, which constitute two distinct fluid compartments within the inner ear (1, 26). This delicate balance is sustained by a highly coordinated system of ion channels, ion transporters, and aquaporins (AQPs), a family of transmembrane proteins that facilitate water transport across cell membranes (27, 28). Endolymph, which is characterized by high potassium and low sodium concentrations, is primarily produced by the stria vascularis, a vascularized epithelial structure in the cochlea. In contrast, endolymph reabsorption predominantly occurs in the endolymphatic sac, a specialized structure responsible for regulating endolymph volume and clearing metabolic waste products (1).

Clinical pathological studies have consistently reported structural and functional abnormalities of the endolymphatic sac in patients with MD, including congenital dysplasia, fibrosis secondary to inflammation, and degenerative changes of epithelial cells associated with advanced age (6, 29, 30). These abnormalities directly impair the reabsorptive capacity of the sac, leading to reduced clearance of endolymph and subsequent fluid accumulation, which represents a key structural determinant of EH formation. At the molecular level, AQPs play a central role in mediating transmembrane water movement within the inner ear, with aquaporin 2 (AQP2) emerging as a critical regulator of endolymph volume (31–37). AQP2 is highly expressed in the epithelial cells of the endolymphatic sac and in vestibular end organs, where it directly modulates water reabsorption by facilitating water flux across the apical membrane of epithelial cells.

Both animal models of EH and clinical studies in patients with MD have linked abnormal AQP2 expression or function to the development of this hydrops. For instance, in experimental rodent models of EH induced by surgical obstruction of the endolymphatic duct, AQP2 expression in the endolymphatic sac is markedly downregulated, consistent with impaired water reabsorption and the pathological accumulation of endolymph (34–36). These findings indicate that AQP2-mediated water transport may represent a potential regulatory mechanism involved in EH; however, whether modulation of AQP2 is sufficient to prevent or reverse MD-associated pathology remains unclear. Therefore, interventions targeting aquaporin function represent a promising but still exploratory therapeutic direction.

### 2.2 Inflammatory and immune axes: cytokines, autoimmunity, and oxidative stress

A substantial and growing body of clinical and preclinical evidence supports the hypothesis that MD may represent a chronic, indolent inflammatory or immune mediated disorder of the inner ear (38–43). Clinically, a subset of patients with MD report that acute vertigo episodes are preceded by upper respiratory tract infections, suggesting that viral exposure may trigger immune activation and subsequent inflammatory injury in vulnerable inner ear tissues (43). Further supporting an autoimmune component, circulating autoantibodies against inner ear specific antigens have been detected in approximately thirty percent of patients with MD (44, 45). Additionally, MD frequently coexists with systemic autoimmune diseases, including rheumatoid arthritis (46–48) and systemic lupus erythematosus (49, 50), further reinforcing the role of immune dysregulation in MD pathogenesis.

At the molecular level, proinflammatory cytokines are key mediators of inner ear inflammation in MD. Specifically, levels of tumor necrosis factor alpha (TNF-alpha) and interleukin 1 beta (IL-1beta), two potent proinflammatory cytokines, are significantly elevated in the endolymphatic fluid of MD patients and in peripheral blood serum compared to healthy controls (51–54). These cytokines activate canonical inflammatory signaling cascades, including the nuclear factor kappa B (NF-kB) pathway (40) and the mitogen activated protein kinase (MAPK) pathway (20), in inner ear tissues such as the stria vascularis and vestibular sensory epithelia. Activation of these pathways amplifies and sustains inflammation by promoting the transcription of additional proinflammatory genes and by recruiting immune cells to the site of injury (54). Over time, persistent inflammatory signaling disrupts the integrity of the blood labyrinth barrier, a specialized barrier that protects inner ear tissues from systemic immune cells and toxins, alters microcirculatory dynamics, and exerts direct cytotoxic effects on cochlear hair cells and vestibular neurons.

In addition to inflammation, oxidative stress, a state of imbalance between the production of reactive oxygen species (ROS) and the capacity of cellular antioxidant systems, plays a critical role in MD related inner ear injury (55–59). Specifically, tissue injury induced by inflammation and ischemia reperfusion events drive the excessive generation of ROS, including superoxide anions and hydrogen peroxide (56, 60). These reactive species cause significant cellular damage by inducing lipid peroxidation of cell membranes, impairing mitochondrial function, and inducing DNA strand breaks, ultimately triggering apoptotic cell death in inner ear sensory cells and neurons (56). The inner ear is particularly vulnerable to damage induced by ROS due to its high metabolic demand and limited antioxidant capacity. Accordingly, strategies that suppress excessive inflammation, modulate immune activity, and enhance antioxidant defense are critical for arresting or slowing the progressive tissue damage and functional decline associated with MD. Accordingly, therapeutic strategies aimed at regulating inflammatory responses, restoring redox balance, and preserving mitochondrial function represent potential approaches for limiting progressive cochleovestibular injury in MD.

### 2.3 Vascular and microcirculatory dysfunction

The stria vascularis, a highly vascularized epithelial structure in the cochlea, represents one of the most metabolically active tissues in the human body, with a metabolic rate comparable to that of the brain (61–63). Its ability to support active ion transport and maintain the ionic gradient between endolymph and perilymph is strictly dependent on a continuous and adequate blood supply. Disturbances of inner ear microcirculation, whether caused by autonomic nervous system dysregulation, vasospasm of cochlear and vestibular arteries, or microthrombosis, reduce cochleovestibular perfusion and induce localized ischemia and hypoxia (6). These vascular insults directly impair stria vascularis function by reducing the availability of oxygen and metabolic substrates, leading to diminished endolymph production and compromised fluid reabsorption in the endolymphatic sac (62, 63). The net effect of these changes is the exacerbation of EH and the worsening of MD symptoms.

Furthermore, tissue hypoxia induced by ischemia promotes the activation of inflammatory signaling pathways and the generation of ROS, establishing a self reinforcing cycle of vascular injury, inflammation, and oxidative stress that perpetuates inner ear damage (56). Given the central role of microcirculatory dysfunction in MD pathogenesis, agents that improve inner ear microcirculatory function have emerged as important therapeutic candidates. For example, betahistine, a histamine H1 agonist and H3 antagonist, has been shown in preclinical studies to dilate cochlear and vestibular arterioles, enhance regional blood flow, and reduce vascular resistance in the inner ear (64–66). Similarly, phytotherapeutic agents such as Ginkgo biloba extract have been reported to improve microcirculation by inhibiting platelet aggregation and enhancing endothelial nitric oxide production, thereby increasing cochlear blood flow and restoring vascular homeostasis (67). Further well-designed clinical studies are required to clarify the contribution of vascular mechanisms and determine whether microcirculation-targeted interventions provide meaningful clinical benefits.

### 2.4 Neuronal injury and apoptosis of auditory and vestibular tissues

The progressive hearing loss and vestibular dysfunction that characterize MD reflect the cumulative and irreversible injury to inner ear sensory epithelia and spiral ganglion neurons. Multiple pathological processes contribute to this neuronal and sensory cell damage, with excitotoxicity and mechanical stress induced by EH being primary drivers (68, 69). Specifically, the increased endolymph volume associated with EH exerts mechanical pressure on the vestibular sensory epithelia and the organ of Corti in the cochlea, disrupting the structure and function of hair cells (16). Additionally, alterations in endolymph ionic composition induced by EH disrupt the electrochemical gradient required for normal neuronal signaling, leading to the excessive release of glutamate, a major excitatory neurotransmitter in the inner ear (42). Experimental studies suggest that excessive glutamate signaling may activate NMDA and AMPA receptors on spiral ganglion neurons and vestibular ganglion neurons, potentially contributing to calcium overload, protease activation, and neuronal stress. However, direct evidence confirming the extent of excitotoxic neuronal degeneration in human MD remains limited (42).

Concurrent inflammation and oxidative stress further contribute to neuronal and sensory cell damage by compromising mitochondrial integrity (59). Specifically, proinflammatory cytokines and ROS induce mitochondrial membrane depolarization, inhibit ATP production, and promote the release of cytochrome c from the mitochondrial intermembrane space into the cytoplasm (59). Cytochrome c then binds to apoptotic protease activating factor 1 (Apaf1) and procaspase 9, forming the apoptosome complex to activate downstream effector caspases (70). These caspases cleave key cellular proteins, driving programmed apoptotic degeneration of cochlear and vestibular neurons and hair cells.

Given the irreversible nature of neuronal and hair cell loss in MD, therapies that target these pathological processes hold significant neuroprotective potential. For instance, agents that reduce glutamate excitotoxicity could prevent calcium overload and neuronal death. Similarly, antioxidants that neutralize ROS and stabilize mitochondrial function may mitigate damage induced by oxidative stress. Additionally, inhibitors of apoptotic signaling could block the final common pathway of cell death (58, 71). While many of these agents are still in preclinical development, their potential to delay or prevent the progressive auditory and vestibular decline that characterizes advanced MD makes them a priority for future clinical research.

## 3 TCM interventions in MD

TCM provides a holistic and multitargeted therapeutic paradigm for the management of MD (21). Unlike conventional approaches that often focus on a single molecular pathway, TCM emphasizes the restoration of systemic balance through syndrome differentiation and multimodal interventions. The current evidence base reflects a distinctive asymmetry. Acupuncture has generated a relatively extensive body of clinical research, including systematic reviews and meta-analyses, although the methodological rigor of many studies is inconsistent and the underlying mechanisms remain incompletely clarified (22, 72–74). Conversely, clinical investigations of herbal therapies are fewer, but preclinical and pharmacological studies of bioactive constituents and molecular pathways are comparatively more advanced. Recognition of this uneven evidence base is essential for accurately evaluating the therapeutic role of TCM and for guiding future research strategies that integrate clinical validation with mechanistic insights. Figure 2 summarizes the principal TCM interventions and their proposed mechanisms.

**Figure 2:**
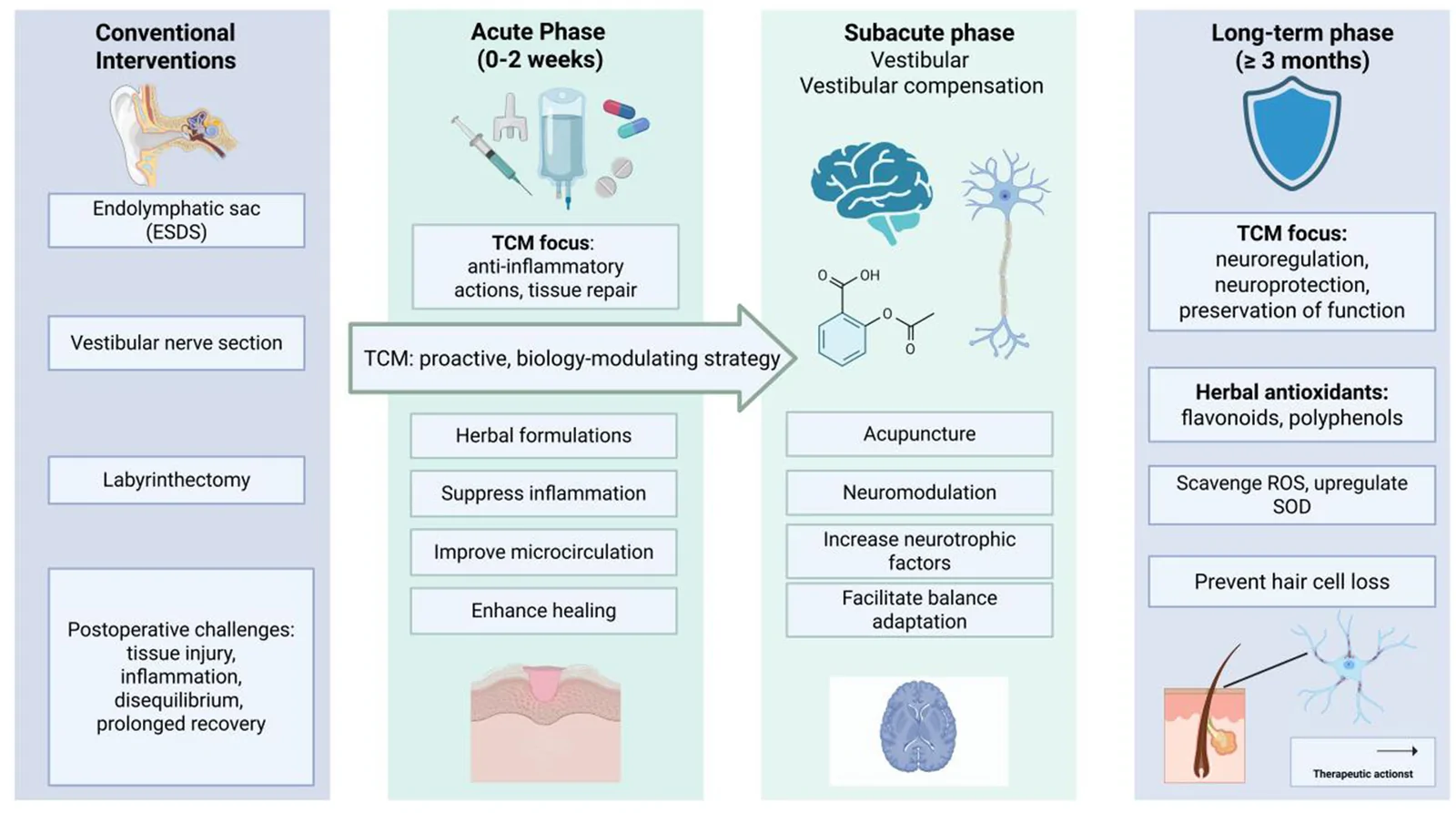
Conceptual framework and phased strategy for integrating Traditional Chinese Medicine (TCM) into postoperative otologic care. Left panel lists common surgical options for refractory Menière's disease—endolymphatic sac decompression, vestibular nerve section, and labyrinthectomy—and associated postoperative challenges, including tissue injury, inflammation, edema, and acute unilateral vestibular loss requiring central compensation. Central panels depict time-dependent TCM actions. Acute phase (0–2 weeks): anti-inflammatory and pro-repair effects; circulation-promoting formulas and supportive therapies reduce mediator release, relieve edema, and improve microcirculation to protect inner ear structures. Subacute phase: enhancement of central vestibular compensation and neuroplasticity via neuromodulatory interventions such as acupuncture. Long-term maintenance (≥3 months): neuroprotection through antioxidant, antiapoptotic herbal constituents that scavenge reactive oxygen species and bolster endogenous defenses. Arrows indicate a synergistic, proactive biology-modulating role for TCM as a perioperative adjunct.

### 3.1 Pathogenetic concepts from the perspective of TCM

In TCM theory, the major manifestations of MD, including vertigo, tinnitus, and hearing loss, are classified under the vertigo syndrome. The term xuan describes visual disturbances such as blurring or darkening of vision, whereas yun refers to vertigo or a sense of rotation. These symptoms are attributed to disharmonies in the zang fu organs, particularly the liver, spleen, and kidney, which disrupt the balance of qi, blood, and yin yang equilibrium. Among the pathogenic constructs, phlegm dampness obstruction is regarded as the most common and clinically significant mechanism. Classical theory holds that spleen deficiency impairs fluid metabolism and leads to the generation of phlegm. The accumulated phlegm and dampness ascend to obscure the sensory orifices, thereby producing vertigo. The maxim stating that vertigo cannot occur without phlegm encapsulates this view and forms the rationale for numerous antiphlegm herbal prescriptions. Other pathogenic mechanisms frequently implicated in MD include hyperactive liver yang manifesting as internal wind, deficiency of kidney essence, and stagnation of qi and blood. Based on these concepts, TCM therapeutic strategies are designed to resolve phlegm and eliminate dampness, pacify liver yang, nourish kidney essence, and promote blood circulation, with treatment tailored to the specific syndrome pattern of the patient through herbal formulas and acupuncture point selection.

### 3.2 Herbal medicine

Herbal therapy represents a central modality of TCM in the treatment of MD. The therapeutic rationale is based on the classical hierarchical formulation principle (jun chen zuo shi), which emphasizes coordinated interaction of multiple herbs to achieve synergistic effects across diverse pathophysiological pathways.

Banxia Baizhu Tianma decoction is a representative formula historically attributed to the spleen and stomach school of Li Dongyuan and later adapted in clinical practice. Within this mixture, *Pinellia* (banxia) dries dampness and resolves phlegm, *Gastrodia* (tianma) pacifies liver wind and alleviates vertigo, *Atractylodes* (baizhu) and *Poria* (fuling) strengthen the spleen and drain dampness, and Chenpi regulates qi and supports phlegm transformation. This formula is widely used for wind phlegm vertigo and has been applied in conditions such as MD, hypertensive vertigo, and vertebrobasilar insufficiency (75–79). Wendan decoction, a classical Tang dynasty prescription, addresses phlegm heat disturbing the clear orifices and is indicated for vertigo with manifestations of phlegm heat. Zexie Tang combines *Alisma* (zexie) with *Atractylodes* (baizhu) to resolve fluid accumulation, traditionally used for disorders of water retention accompanied by vertigo (80), a concept that closely parallels modern notions of endolymphatic imbalance.

Several clinical studies suggest that herbal formulas may alleviate symptoms of MD. For example, modified Banxia Baizhu Tianma decoction demonstrated an overall response rate of ninety-four point seventy-four percent in one study, while integrated manipulative and herbal interventions showed rapid symptom improvement (81). Despite these preliminary observations, the majority of trials are limited by small sample sizes, inadequate placebo or sham controls, lack of blinding, reliance on subjective endpoints, and short follow-up periods. The fluctuating natural course of MD and the substantial placebo response, which can reach reductions in vertigo attack frequency of approximately fifty-two point five percent in placebo cohorts, further complicate efficacy assessments (82). Therefore, although initial results are suggestive, the field requires rigorously designed randomized controlled trials with standardized interventions, objective outcome measures, sufficient statistical power, and long-term follow-up to generate reliable evidence.

Pharmacological studies have identified several bioactive constituents with potential relevance for MD. Gastrodia elata contains gastrodin, which exerts anticonvulsant, sedative, and neuroprotective effects, and may enhance microcirculation in cerebral and cochlear tissues (83). Pinellia ternata provides alkaloids with antiemetic properties, beneficial for controlling nausea and vomiting during acute MD attacks (84). Alisma species yield triterpenoids with diuretic activity that facilitate fluid excretion, conceptually analogous to conventional diuretic therapy in MD (85). Ginkgo biloba extracts, rich in flavonoids and terpene lactones, improve microcirculation, reduce oxidative stress, and scavenge free radicals, and are widely used for tinnitus and vertigo (67, 86, 87). These mechanistic insights, derived largely from preclinical models, align with contemporary understanding of MD pathophysiology involving fluid balance, inflammation, microcirculatory impairment, oxidative stress, and neuronal injury. However, direct clinical translation requires cautious interpretation, as human evidence remains limited.

### 3.3 Acupuncture

Acupuncture has attracted greater clinical research attention than herbal medicine in the context of MD. Multiple systematic reviews and meta analyses report that acupuncture, either alone or in combination with conventional therapy, improves both subjective symptoms and objective auditory outcomes. A recent meta analysis of six randomized controlled trials published in 2024 found that acupuncture alone or acupuncture combined with conventional therapy significantly outperformed conventional therapy alone for overall efficacy (22). Additional pooled analyses demonstrated reductions in Dizziness Handicap Inventory scores, improvements in Tinnitus Handicap Inventory scores, decreased ear fullness measured by visual analog scale, and enhanced pure tone audiometry thresholds (22).

Together, these findings suggest that acupuncture may exert beneficial effects on both the perceived burden of disease and measurable auditory function. However, the evidence base remains constrained by methodological limitations, including small sample sizes, variability in acupuncture protocols, and inconsistent use of sham controls. The high placebo responsiveness of MD further underscores the need for caution, as nonspecific effects could contribute to the observed benefits. To advance the field, future research should prioritize multicentre randomized controlled trials with standardized interventions and objective outcomes, coupled with translational investigations to validate mechanistic hypotheses such as the roles of aquaporins, inflammatory signaling, microcirculatory regulation, and neuroprotection. Rigorous quality control of herbal materials and standardized reporting of acupuncture protocols will also be essential. An integrated strategy combining clinical and mechanistic evidence generation is required to define the role of TCM in evidence-based, personalized management of MD.

## 4 Pharmacological and physiological mechanisms of TCM in Menière's disease

### 4.1 Regulation of inner ear fluid homeostasis through aquaporins and ion transport

In TCM theory, the conceptual frameworks of phlegm and dampness describe the pathological accumulation and impaired kinetics of biological fluids. This traditional notion corresponds closely with the definitive pathophysiological hallmark of MD, namely EH. Contemporary experimental investigations have provided direct molecular explanations validating this traditional paradigm. A seminal preclinical study demonstrated the capacity of Wendan decoction (WDT), a classical herbal formula, to modulate inner ear fluid equilibrium (20). In that investigation, guinea pigs were subjected to surgical induction of EH, successfully reproducing core pathological features of MD including labyrinthine distention and hair cell injury. Administration of WDT significantly improved neurovestibular function compared with untreated controls. Histological analysis confirmed a marked reduction in labyrinthine hydrops and the structural preservation of outer hair cells.

Crucially, mechanistic assays revealed that WDT downregulated the expression of AQP2 in inner ear tissues (20). This experimental finding represents a notable translational observation because it directly links a classical phlegm-resolving prescription with the molecular regulation of water transport proteins within the membranous labyrinth. Furthermore, these findings provide a possible biological interpretation of the traditional TCM concept of phlegm-dampness in relation to fluid homeostasis, although they should not be regarded as direct pharmacological validation of classical TCM theories. These collective results, obtained from animal models, suggest multiple bioactive compounds within herbal formulations may act synergistically to modulate the transcriptional and posttranscriptional expression of aquaporins, thereby restoring endolymph absorption, mitigating EH, and ultimately alleviating clinical vertigo.

### 4.2 Anti-inflammatory effects

Inflammation and immune dysregulation are increasingly recognized as central upstream drivers of MD pathogenesis. An expanding repertoire of TCM formulas and isolated botanical constituents have demonstrated antiinflammatory and immunomodulatory effects in preclinical models, targeting the core inflammatory pathways that perpetuate inner ear damage. The aforementioned evaluation of Wendan decoction (WDT) demonstrated a significant capacity to suppress localized tissue inflammation. Utilizing network pharmacology integration coupled with *in vivo* validation, researchers established that WDT inhibited the activation of the mitogen activated protein kinase (MAPK) pathway and reduced the downstream release of proinflammatory cytokines (20, 88). Given the central role of the MAPK pathway in governing cellular inflammation, proliferation, and programmed cell death, its targeted inhibition provides a plausible mechanism through which TCM interventions can mitigate chronic inner ear injury.

Other botanical interventions provide converging evidence validating this therapeutic axis. For example, Ginkgo biloba extract has been shown in experimental studies to suppress the synthesis and release of TNF-α and IL-β, both of which represent critical proinflammatory mediators within the cochleovestibular system (89). Parallel to these herbal actions, acupuncture has been reported to exert systemic anti-inflammatory effects, possibly mediated through the modulation of vagal nerve activity and the subsequent activation of the cholinergic anti-inflammatory reflex, which collectively downregulates central and peripheral neuroinflammation. Taken together, these mechanistic hypotheses indicate that TCM deploys multitargeted antiinflammatory mechanisms that could potentially attenuate chronic tissue injury, thereby protecting delicate inner ear structures from immune mediated degeneration. Confirmation in human studies remains necessary.

## 5 Future challenges and perspectives

This review highlights that TCM, particularly acupuncture and selected herbal formulations, shows preliminary therapeutic potential for MD based on the current, limited evidence. Acupuncture is supported by a relatively concentrated body of clinical evidence suggesting improvements in vertigo, tinnitus, aural fullness, and, in some studies, possible changes in hearing thresholds. Preclinical and pharmacological studies of herbal prescriptions such as WDT have identified biologically plausible mechanisms that include modulation of AQP2 expression, suppression of inflammatory MAPK signaling, enhancement of inner ear microcirculation, and mitigation of oxidative stress and apoptosis. Collectively, these findings suggest that TCM provides a multitargeted and system oriented therapeutic strategy that may be relevant for symptom management in MD, although the strength of evidence remains limited (Figure 3).

**Figure 3:**
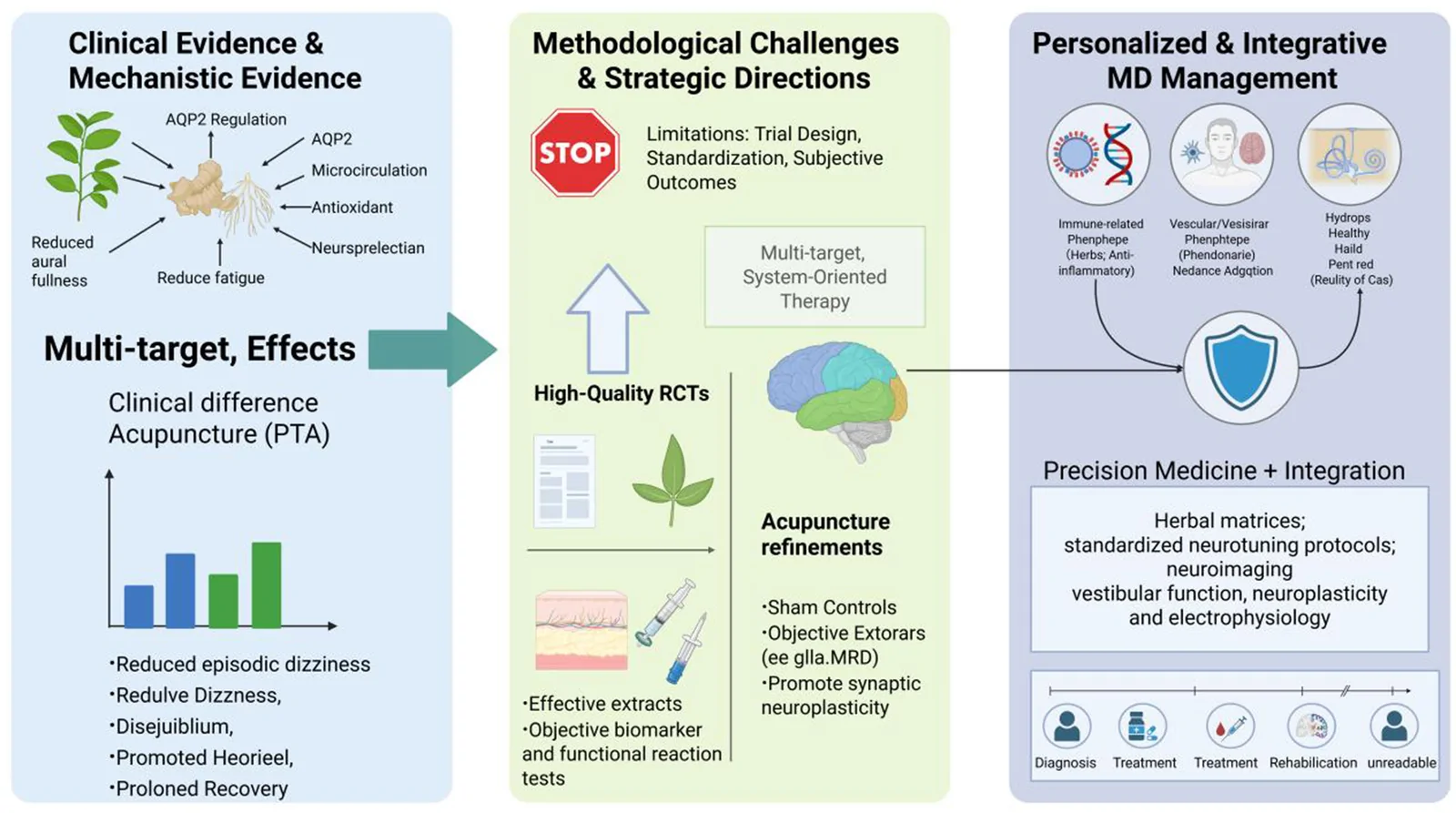
Schematic summary of translational priorities for Traditional Chinese medicine (TCM) in Menière's disease (MD). The left panel synthesizes clinical and mechanistic evidence linking TCM targets—aquaporin-2 regulation, improved microcirculation, anti-inflammatory and neuroprotective actions—to symptomatic gains observed with acupuncture and selected herbal formulas. The central panel highlights methodological hurdles and strategic directions: trial design limitations, need for standardization, objective biomarkers and high-quality randomized controlled trials, plus refinement of acupuncture methodology to include rigorous sham controls and neurophysiological end points. The right panel projects a personalized, integrative care model that combines phenotype-guided TCM (immune, vascular, hydrops-predominant profiles) with precision diagnostics, standardized herbal and neuromodulatory protocols, neuroimaging and electrophysiology, and coordinated rehabilitation. Bidirectional arrows indicate reciprocal translation between mechanistic research and clinical practice. This framework emphasizes multi-target, system-oriented interventions and the imperative to validate TCM approaches with objective outcomes to enable precision integrative management of MD.

Despite these advances, several challenges persist. The overall quality of existing evidence remains uneven, with substantial variability in study design, intervention consistency, and outcome measurement. Mechanistic research, although increasingly sophisticated, is not yet adequate to support broad clinical translation. Addressing these limitations will require coordinated efforts across multiple domains. For herbal medicine, large-scale, rigorously designed, multicentre, double-blind, placebo-controlled randomized trials are urgently needed. Priority should be given to formulations with strong mechanistic foundations, such as WDT and Banxia Baizhu Tianma decoction. These trials must adhere to international standards, specifically the Consolidated Standards of Reporting Trials (CONSORT) guidelines, to ensure methodological transparency and reproducibility. Standardization and quality control of herbal extracts and preparations should be strengthened to minimize batch variability and increase clinical reliability. Future studies should incorporate objective biomarkers in addition to conventional symptom scales, including gadolinium enhanced delayed inner ear magnetic resonance imaging (MRI) for assessing EH, comprehensive audiometry, and vestibular function tests such as the video head impulse test (vHIT) and vestibular evoked myogenic potentials (VEMPs) (12, 90–93). For acupuncture, similar methodological refinement is essential. Upcoming trials should employ carefully designed sham acupuncture procedures with assessment of blinding effectiveness to differentiate specific therapeutic effects from nonspecific responses. Development of standardized and evidence based treatment protocols, including acupoint selection, stimulation parameters, and treatment duration, will help reduce heterogeneity and improve comparability across studies. Mechanistic investigations should also be expanded through the integration of advanced neuroimaging and electrophysiological approaches, including functional MRI and electroencephalography, to clarify how acupuncture modulates vestibular pathways, autonomic activity, and central sensory integration (94, 95). To more fully address the precision medicine perspective, future research must also focus on molecular phenotypes, inflammatory endotypes, and genetic stratification in MD (96). Emerging data suggest that patients may exhibit distinct biological profiles, including autoimmune-associated, vascular-ischemic, or inflammatory phenotypes. In contrast, EH detected by MRI should be regarded as a structural imaging feature rather than an etiological subtype. These biological profiles and imaging characteristics may provide complementary information for patient stratification. These endotypes may respond differently to specific TCM interventions. For instance, patients with elevated proinflammatory cytokines could be candidates for herbal formulas with robust anti-inflammatory activity, whereas those with microcirculatory impairment might benefit from blood-circulation-promoting herbs or acupuncture. Furthermore, genetic polymorphisms affecting ion channels or aquaporin regulation, as well as imaging biomarkers derived from advanced MRI sequences, may aid in matching individual patients to the most appropriate TCM regimen. This stratification aligns with the TCM principle of syndrome differentiation and represents a tangible pathway toward precision-based integrative care.

The longitudinal aim is to establish an integrative clinical model in which TCM is evaluated not as an alternative but as a scientifically testable adjunctive modality targeting defined pathophysiological mechanisms. In such a model, TCM can be incorporated throughout the continuum of care, from diagnosis and conservative management to perioperative support and chronic rehabilitation. For example, patients with prominent inflammatory signatures may benefit preferentially from herbal formulations possessing strong antiinflammatory activity (52, 97–99), whereas individuals undergoing vestibular ablative procedures may achieve faster central compensation when perioperative acupuncture is integrated into their rehabilitation program (100–102). By combining the precision of modern biomedicine with the holistic and multitargeted regulatory effects of TCM, this integrative strategy has the potential to deliver more comprehensive and individualized management for MD, provided that the necessary rigorous clinical validation is achieved.

## 6 Conclusion

This review synthesizes current clinical and preclinical evidence regarding the potential role of TCM in the management of MD. Endolymphatic hydrops remains the central pathophysiological substrate of MD, and convergent mechanisms involving dysregulated fluid homeostasis, inflammatory and immune imbalance, microcirculatory impairment, and neuronal injury provide a biologically plausible foundation for therapeutic intervention. Within this context, TCM interventions exhibit distinct profiles: acupuncture is supported by a body of clinical studies suggesting symptomatic improvement and, in some investigations, audiometric benefits, whereas herbal prescriptions have demonstrated compelling mechanistic effects in experimental models, including regulation of AQP2 expression, inhibition of NF-κB and MAPK-mediated inflammatory signaling, enhancement of inner ear microcirculation, and attenuation of oxidative stress and apoptosis. However, the available clinical evidence is largely preliminary, with most studies limited by small sample sizes, lack of placebo controls, and methodological heterogeneity. Moreover, the substantial placebo response in MD necessitates cautious interpretation of uncontrolled observations.

Given these limitations, the current evidence is insufficient to support TCM as a core therapy for MD. Instead, TCM may serve as a complementary approach for symptom management in selected patients, but its clinical application remains investigational and requires further validation. To advance the field, several strategic priorities must be addressed. First, well-designed, adequately powered, multicentre randomized controlled trials with appropriate control conditions and standardized outcome measures are needed to establish the clinical efficacy of TCM interventions. Second, greater standardization and quality control of herbal preparations are required to improve reproducibility and facilitate translational application. Third, acupuncture research requires more rigorous methodological frameworks, including carefully designed sham acupuncture procedures with assessment of blinding effectiveness and standardized treatment protocols. Finally, integrated translational studies linking molecular, immunological, and imaging biomarkers with clinically meaningful outcomes are needed to clarify the mechanisms and identify potential responders. The adoption of a precision-oriented integrative framework, wherein TCM pattern differentiation is aligned with molecular, immunologic, and imaging-based phenotypes, may provide a future pathway toward individualized therapeutic strategies for MD. With continued methodological refinement and rigorous validation, the therapeutic potential and appropriate clinical positioning of TCM in MD can be better defined; however, firm clinical recommendations should await confirmation from high-quality clinical trials.
